# Parasite microbiome project: Grand challenges

**DOI:** 10.1371/journal.ppat.1008028

**Published:** 2019-10-10

**Authors:** Nolwenn M. Dheilly, Joaquín Martínez Martínez, Karyna Rosario, Paul J. Brindley, Raina N. Fichorova, Jonathan Z. Kaye, Kevin D. Kohl, Laura J. Knoll, Julius Lukeš, Susan L. Perkins, Robert Poulin, Lynn Schriml, Luke R. Thompson

**Affiliations:** 1 School of Marine and Atmospheric Sciences, Stony Brook University, Stony Brook, New York, United States of America; 2 Bigelow Laboratory for Ocean Sciences, East Boothbay, Maine, United States of America; 3 College of Marine Science, University of South Florida, Saint Petersburg, Florida, United States of America; 4 Department of Microbiology, Immunology and Tropical Medicine, George Washington University, Washington, DC, United States of America; 5 Research Center for Neglected Diseases of Poverty, School of Medicine & Health Sciences, George Washington University, Washington, DC, United States of America; 6 Genital Tract Biology Division, Department of Obstetrics, Gynecology and Reproductive Biology, Brigham and Women’s Hospital, Harvard Medical School, Boston, Massachusetts, United States of America; 7 Gordon and Betty Moore Foundation, Palo Alto, California, United States of America; 8 Department of Biological Sciences, University of Pittsburgh, Pittsburgh, Pennsylvania, United States of America; 9 Department of Medical Microbiology and Immunology, University of Wisconsin-Madison, Madison, Wisconsin, United States of America; 10 Institute of Parasitology, Biology Centre, Czech Academy of Sciences and Faculty of Sciences, University of South Bohemia, České Budějovice (Budweis), Czech Republic; 11 Sackler Institute for Comparative Genomics, American Museum of Natural History, New York, New York, United States of America; 12 Department of Zoology, University of Otago, Dunedin, New Zealand; 13 Department of Epidemiology and Public Health, University of Maryland School of Medicine, Baltimore, Maryland, United States of America; 14 Department of Biological Sciences and Northern Gulf Institute, University of Southern Mississippi, Hattiesburg, Mississippi, United States of America; 15 Ocean Chemistry and Ecosystems Division, Atlantic Oceanographic and Meteorological Laboratory, National Oceanic and Atmospheric Administration, La Jolla, California, United States of America; University of Utah, UNITED STATES

The first Parasite Microbiome Project (PMP) Workshop (January 9–14, 2019, Clearwater, Florida, United States) hosted researchers from across continents and disciplines to lay the foundation of the PMP consortium. The PMP vision is to catalyze scientific discourse and explorations through a systems approach, toward an integrated understanding of the microbiota of parasites and their impact on health and disease. The participants identified knowledge gaps and grand challenges in the field of host–parasite–microbe interactions summarized here. The PMP will provide an interactive centralized platform and resource for transdisciplinary collaboration to propel the field of parasitology forward by disentangling complex interactions between parasites and hosts, their respective microbiota, and microbial communities in the parasite’s direct environment (Fig 1).

**Fig 1:**
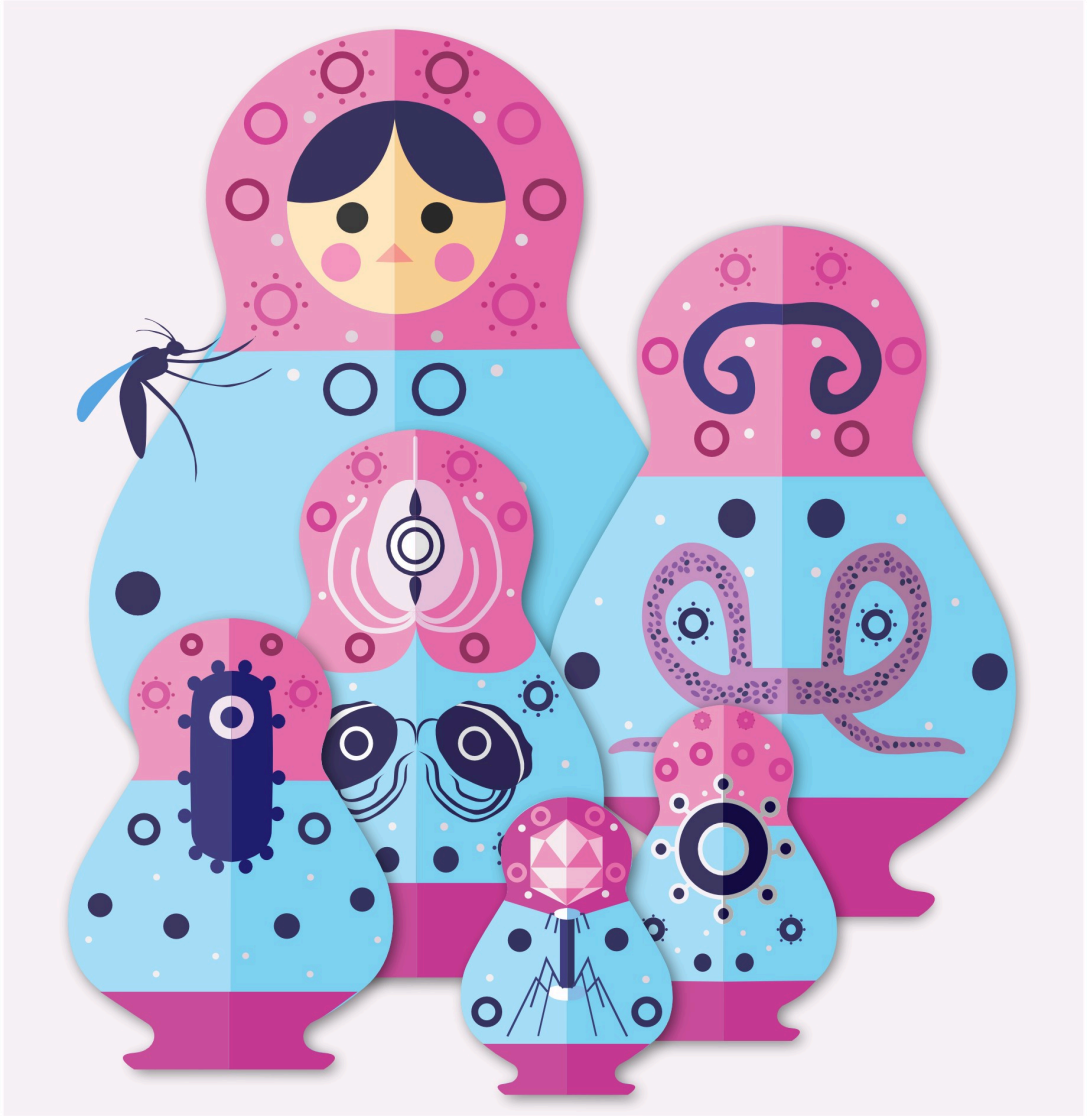
The complex nature and interrelations of host–parasite–microbe interactions are illustrated using a Matryoshka (Russian nesting) doll metaphor. The PMP aims to elucidate nested interactions between a given host and parasites (e.g., helminths and protists) that are themselves hosts to their own symbionts and parasites (e.g., viruses and bacteria). Artwork by Meredith Brindley (http://meredithbrindley.com/). PMP, Parasite Microbiome Project.

Parasitism is a successful lifestyle that has evolved in virtually every clade of multicellular organisms [1–3] and protists [4, 5]. Parasitology seeks to develop the means to prevent, limit, or cure infections by parasites for the benefit of humans, agriculture, and wildlife and to understand how parasitism and parasitic disease impact not only the host but also host communities and ecosystem health. This is a challenging task, considering the diversity and complex nature of host–parasite interactions. Parasitic organisms harbor a rich tapestry of traits associated with survival and must navigate the host immune response to reproduce and be effectively transmitted to the next host.

An improved understanding of underlying molecular mechanisms and evolutionary patterns that explain interindividual, temporal, and geographic variation in the outcomes of parasitic infections is much needed [6–9]. There is an increasing recognition of the potential for host- and parasite-associated microbiota―endo- and/or ectosymbiotic archaea, bacteria, viruses, and micro-eukaryotes―to influence and shape host–parasite interactions [10]. In the past few years, the concept of individuality has given way to that of “holobiont” with the recognition that each organism is a composite of organisms [11–14] (Fig 1, Box 1). Yet we have limited insight into the nature and importance of these interactions for parasite ecology and evolution [15, 16], and not a single parasite species has its entire microbiome fully characterized.

**Box 1. Key microbiome and holobiont concepts applied to parasitology**

**Direct environment:** environment of the parasite at the time of sampling (host-associated and free-living stages).

**Parasite-associated microbiome:** collection of the genomes of the microbiota (viruses, bacteria, archaea, and micro-eukaryotes) that are either chromosomally integrated or episomal, intracellular, or attached to the surface of the parasite.

**Host-associated microbiome:** collection of the genomes of the microbiota that are associated with the host, either in the direct environment of the parasite, or in a distant tissue or anatomic compartment of the host.

**Environmental microbiome:** collection of the genomes of the microbiota that are present in the direct environment of free-living (encysted or mobile) life stages of parasites.

**Holobiont:** a unit of biological organization composed of a host and its microbiota, inclusive of transient and persistent microbes.

**Hologenome:** the complete genetic content of an organism’s genome, including nuclear and organellar genomes, and its microbiome.

## The PMP

**Fig 2:**
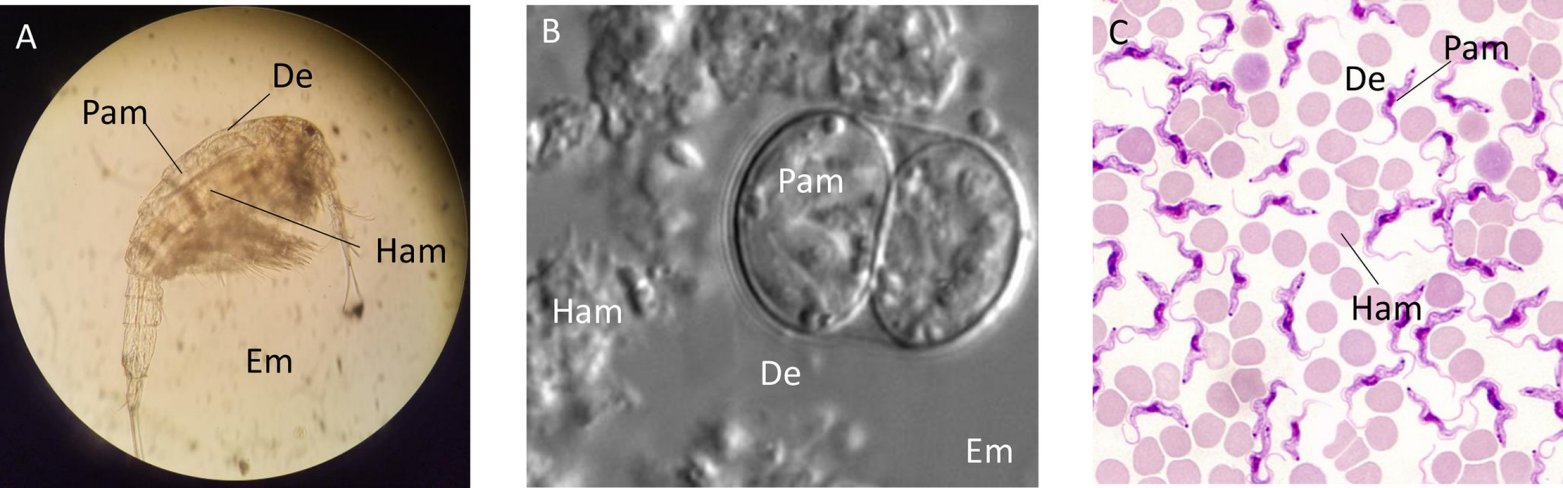
Examples conveying context-dependent usage of the microbiome and holobiont concepts in parasitology. (A) Procercoid stage of the cestode *Schistocephalus solidus* in the body cavity of a cyclopoid copepod. The Pam may be collected from procercoids. The De is the body cavity of the copepod. The Ham may be collected from the gut or other host tissues whereas the Em may be collected from the water. (B) Oocyst of *Toxoplasma gondii* that sporulated upon excretion with cat feces. The Pam may be collected from purified oocysts. Distinction between the Ham, De, and Em is difficult. (C) *Trypanosoma* sp. among red blood cells. The Em is not represented. The intracellular microbes potentially present within red blood cells may be considered Ham whereas microbes within the plasma can be considered in the De of the parasite. *Image credits*: *M*. *Hahn; L*. *Knoll and J*. *P*. *Dubey; J*. *Lukeš*. De, direct environment; Em, environmental microbiome; Ham, host-associated microbiome; Pam, parasite-associated microbiome.

**Fig 3:**
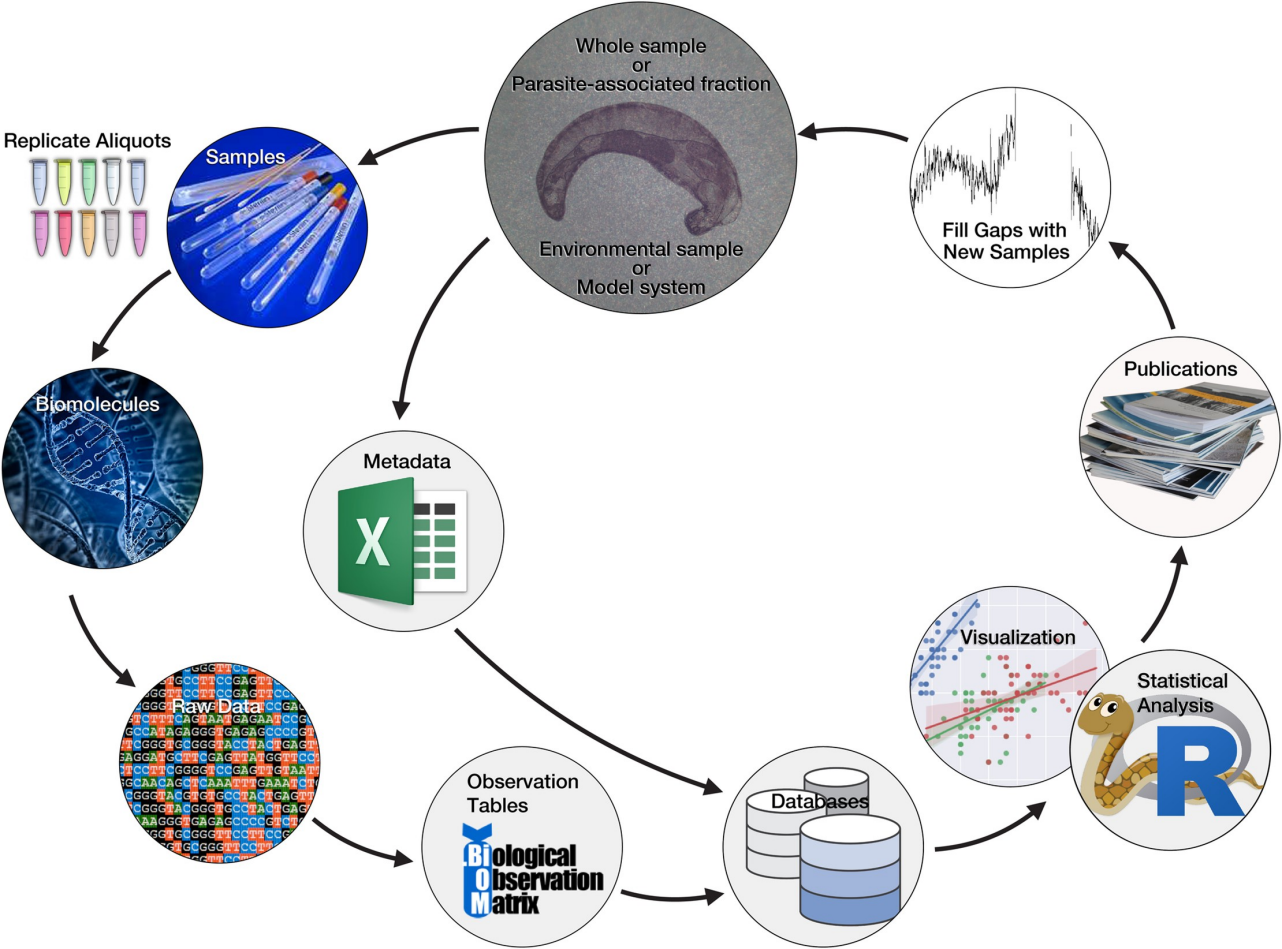
Proposed workflow for processing PMP samples from parasites and host tissues. PMP, Parasite Microbiome Project.

The PMP envisions a holistic understanding of host–parasite–microbe interactions by fostering global transdisciplinary explorations of the microbiomes of parasites and their direct environment (Fig 2; Box 1) [17]. The PMP will be enabled by new and existing tools, technologies, and standards developed for microbiomes and tailored for analyses of host–parasite–microbe interactions. Areas of focus will include (1) development of relevant standards for metadata collection and curation, (2) methods development for processing of parasite-associated microbes, (3) multi-omics technologies, and (4) tailoring of analytical tools for parasite-associated microbiome research. Methods and data will be shared freely with the scientific community and public using open data standards. Establishing and optimizing these methods will initially require a “test” collection of well-characterized parasite isolates/models and a comprehensive identification of parasite-associated microbes. The PMP will establish a workflow and centralized platform to maximize parasite sampling efforts and facilitate parasite microbiome research for the community at large (Fig 3; Table 1).

**Table 1:** Methods to tackle the grand challenges of parasite microbiome research.

Method	Challenge and/or proposed approach	Reference
***Sample collection***		
• Metadata collection	Must be complete and standardized; collect adhering to MIxS environmental package for parasite-associated samples	[18–20]
• Environmental parasite microbiota	Need to fractionate samples to distinguish parasite-associated microbiome from direct environment microbiome; freezing and/or preservation in ethanol or RNAlater depending on downstream processing	
• Laboratory parasite microbiota	Need growth conditions, in vitro animal model systems, e.g., tissue, organoids, cell lines	[21, 22]
***Molecular characterization***	*** ***	*** ***
• Metagenomic DNA sequencing	Capture whole community including prokaryotes, micro-eukaryotes, and abundant or actively replicating viruses	
• Amplicon DNA sequencing	Group-specific taxonomic profiling of key groups	
• Viral community sequencing	Viral purification (viral metagenomes) or sequencing of vSAGs	[20, 23–25]
• Parasite genome sequencing	Need to supplement reference genomic databases and identify role of host genotype in shaping the interactions of resident microbes	[26, 27]
• Transcriptomics, cDNA metagenomics	Detection of RNA viruses	[28]
• Metabolomics	Mass spectrometry (LC-MS/MS, GC-MS)	
• Microscopy for spatial organization	FISH and microscopy to identify localization of microbes on/inside parasite and in relation to each other; microscopy of living parasites to reveal temporal patterns	[29, 30]
***Data analysis***		
• Data mining	Search existing sequence archives and parasite sequencing projects for parasite microbiomes	
• Reference databases	Build upon existing databases (e.g., EuPathDB)	https://eupathdb.org
• Genome assembly	Need to assemble microbial genomes from metagenomes in context of host and parasite genomic DNA; also assemble parasite genomes	
• Metagenomic taxonomic and functional analysis	Taxonomic composition using nucleotide composition (e.g., Kraken, Nonpareil) and marker genes (e.g., MetaPhlAn) and species-specific fuctional composition using nucleotide and protein databases (e.g., HUMAnN2)	[31–34]
• Amplicon analysis	Database curation and exact-sequence methods	[35–39]
• Multi-omics analysis	Compare profiles of taxa, genes, metabolites across multi-omics methods	
***Data sharing***		
• Protocols	Protocols for sample collection, processing, and analysis; share on protocol-sharing service (e.g., Protocols.io)	https://protocols.io
• Code	Processing and analysis code; share on GitHub repository and permanent archive (e.g., Zenodo)	https://github.com, https://zenodo.org, http://gensc.org
• Study metadata	Study title, description, design, points of contact, and publication DOI; share on GitHub repository and permanent archive (e.g., Zenodo)	
• Sample metadata	MIxS-compliant metadata (see above); share on GitHub repository and permanent archive (e.g., Zenodo)	
• Raw data	All raw data after collection; deposit in EBI, GenBank, and other data archives	https://www.ebi.ac.uk

**Abbreviations:** DOI, digital object identifier; EBI, European bioinformatics institute; FISH, fluorescence in situ hybridization; GC-MS, gas chromatography-mass spectrometry; LC-MS/MS, liquid chromatography-tandem mass spectrometry; MIxS, Minimal Information about any Sequence; vSAG, viral single amplified genome

A primary advantage of a centralized platform like the PMP is the collation of large aggregates of associated metadata that can be harnessed to uncover, and eventually understand, patterns of microbial diversity and ecology [40, 35]. Therefore, detailed metadata associated with each study and sample are absolutely critical to maximize the utility of each. To facilitate future research opportunities, the PMP will encourage tracking of metadata connected to both the sample and its processing and the deposition of host and parasite vouchers into museum collections to allow future analysis opportunities when new techniques and hypotheses arise [41, 42]. Any additional tissues and extracted biomolecules should also be maintained in dedicated (cryo-) collections. We will adapt practices and lessons learned by the Earth Microbiome Project (EMP) [35, 43], e.g., preparation of multiple (homogenous) aliquots of the samples to be studied [44], in a manner that is best suited for the PMP. By providing tested methods and developing standards for parasite microbiome research, the PMP foresees the following:

- Elevating the integral role of the microbiome in host–parasite ecology and evolution (in a dynamic environment) to promote solution-oriented research in parasitic disease management.
- Steering the larger microbiome community towards analysis of the micro-eukaryotic component of the microbiome.
- Building an inclusive and transdisciplinary PMP community that catalyzes analysis of natural and model parasite systems.
- Becoming a community hub that coordinates discussions fostering collaborative research to address current and future grand challenges.

## Grand challenges

We encourage the scientific community to join the PMP in addressing grand challenges in the field of host–parasite–microbe interactions and designing creative experiments in a diversity of systems to explore the areas outlined next.

### Identifying core and transient parasite-associated microbes

Parasite-associated microbiomes remain largely unknown, in part due to the inherent difficulties of studying the parasites themselves, e.g., challenging or nonexistent in vitro cultivation systems, complex life cycles, ethical considerations, obligate host environments that are difficult to simulate in experimental models, and national borders. Another specific challenge for investigating microbial communities associated with parasites is the necessity to isolate the parasite from the background sampled material and from host-associated microbes. The microbiota within a parasite can be divided into core microbes (intrinsic to the parasite or at least to a specific parasitic life stage) and transient microbes (temporarily acquired by the parasite from its direct environment). Comparative analyses between the parasite microbiome, the microbiome of the corresponding infected host, and a control noninfected host from the same environment will be needed to rule out potential microbial contaminants from the direct environment.

Parasite holobiont research will need to ascertain whether microbes are vertically transmitted from parasitic parent to offspring, horizontally transmitted between parasites coinfecting the same host, or transmitted between the parasite and its direct environment (the host or the external environment of the free-living stages). The objective will be to discern to what extent the parasite-associated microbiota is determined by the parasite (maintained across developmental growth, reproduction, and dispersal), by the composition of the microbiota in its direct environment, or by abiotic factors. This objective could be examined, for example, by comparing the microbiomes of parasites (1) infecting multiple host species (for generalist parasites or parasites with complex life cycles), (2) at different life stages, (3) isolated from spatially and temporally separated populations or populations with different diets, or (4) coinfecting the same host.

### Understanding the roles of parasites in microbe evolution and host–microbe interactions

Parasite prevalence in a population, route(s) of parasite transmission, and interdependence between the microbe and its parasitic host will drive the evolution of the microbe’s modes of transmission. These modes are of particular interest because they will drive microbial virulence both for the parasite and its host [45]. Parasites may influence the composition of the microbiota of their hosts by diverse means. For example, parasites may (1) be vectors or reservoirs of microbes; (2) exert pressure on the host during infection, leading to the evolution of defensive microbes; (3) compete with host microbiota for nutrients or provide metabolic and genetic reservoirs to support the growth and survival of other host microbial species; (4) modify the host environment, e.g., pH, to the benefit of other microbes; and/or (5) induce an immune response by the host that, in turn, impacts the host’s microbiome.

The extent to which the host microbiome is determined by its parasites can be investigated by comparing the microbiome of individuals infected by different parasitic species and/or strains [46]. When treatments are available, they can be used to determine whether the host microbiome returns to its original state after removal of the parasite. In addition, characterizing the underlying mechanisms will be necessary to determine whether the parasite directly or indirectly interacts with the host microbiome and whether this is beneficial to the parasite or a side effect of the infection. Furthermore, by serving as vectors or reservoirs of microbes, parasites could alter the evolution of microbes by providing opportunities for host switching or novel microbe–microbe interactions that may lead to genetic exchanges. In order to gain an evolutionary perspective on host–parasite–microbe interactions, evolutionary studies encompassing microbes across host and parasite species are necessary to identify patterns of cospeciation and speciation following host shifts.

### Understanding the functional role of microbes in parasite fitness and host diseases

Parasites and associated microbes can be viewed as a community of organisms that experience different selection pressures, despite the high potential for interdependence. Microbes can be either beneficial (mutualistic) or antagonistic (parasitic or with fitness conflicts) to the parasite. The nature of the interaction would lead to radically different effects of the microbes on the evolution of the holobiont and the host–parasite interaction. Similarly, microbes associated with the host may be beneficial for the parasite, as a result of selection for cooperation, or they may be detrimental due to the competition for nutrients and/or space. The nature of parasite–microbe interactions may have a critical effect on the parasite’s fitness and host disease. For example, viral symbionts of parasitic protists can divert host responses toward antiviral immunity, which is inefficient in clearing the eukaryotic infection and may aid the parasite survival [21].

Understanding the impact of microbes on the fitness of hosts and parasites is of relevance to epidemiological studies and is expected to provide new opportunities for therapeutic interventions. The inherent complexity of the study of host–parasite–microbe interactions necessitates the application of methods from the field of community genetics, wherein it is acceptable that the gene that governs a given phenotype resides in the genome of another species and is dependent on the environment [47]. Here, the environment of the host and parasite is the microbiome, and its impact on the evolution of the system can be tested by measuring parameters of the host and parasite fitness in the presence of different microbes. Alternatively, host–microbe interactions can be tested by considering the host as the environment.

### Identifying patterns and processes of host–parasite–microbe coevolution

Interindividual variations in the outcome of a parasitic infection resulting from variations in host susceptibility, parasite virulence, and host–parasite compatibility can be better understood in the context of the geographic mosaic of coevolution [48]. Microbes also show geographic variation, and they can participate in coevolution by shifting selection pressures away from or towards either the host or the parasite [49–51]. With appropriate experimental systems, geographic variations affecting the role of microbes in host–parasite interactions can be assessed by using a complete cross-experimental design, in which hosts from different localities are infected with parasites from their corresponding localities in the presence of either microbes isolated from the same localities or microbes from different test localities. Identification of temporal variations in selection pressures on microbes involved in host–parasite interactions would require time-shift experiments, wherein the microbes that have evolved with the host and parasite are transferred back to an ancestral host and parasite. Finally, when possible, experimental evolution of parasites and hosts in the presence or absence of the identified microbes can been used to test the effect of specific microbes on the evolution of the system and to identify mechanisms involved in parasite–microbe interaction.

## Moving forward

The PMP consortium proposes a two-phase development, analogous to the Human Microbiome Project (HMP) [52]. Phase one will compile information on previously characterized parasite-associated microbes and parasite–microbe interactions (already partially reviewed in [15–16, 53]), mine genomic and transcriptomic databases to detect microbial sequences, and characterize the complete microbiome of a set of parasites representing diverse taxa and environments. A main focus during this phase will be on preparing a website and developing and sharing best practices, methods, and standards for effective sample management and integration of data. The PMP, in collaboration with the Genomic Standards Consortium (GSC; gensc.org), has initiated the development of a new parasite-associated package to be added to the Minimal Information about any Sequence (MIxS) standard [18]. This package will facilitate the collection, standardization, reporting, and integrated analyses of metadata to capture the parasite microbiome contextual information describing the host, environment, sample and sequencing data. We anticipate the MIxS-PMP to be available by the end of 2019.

The second phase of the project will rely on the development of experimental model systems that may be employed to prove cause-effect relationships between parasite virulence, diseases, and microbiome composition, as well as to investigate the underlying molecular mechanisms and the evolution of host–parasite–microbe interactions. Findings from initial microbiome characterizations during phase one and previously proposed experimental model systems [53] will guide the evaluation and selection of systems most suitable for addressing the scientific grand challenges identified herein.

Given the important role of parasites in ecosystems, human health, and agricultural management, propelling the field of parasitology in a coordinated way with the PMP can have an enormous payoff (Table 2). The PMP will necessitate both significant funding to conduct challenging research as well as engagement from the community to provide high-quality samples and to share detailed and accurate metadata information. Therefore, we propose constituting a community of researchers that meet annually for workshops and symposia. With this opinion article, we invite reader comments to better define grand challenges and research needs moving forward.

**Table 2:** Representative examples of organisms for which uncovering parasite–microbe interactions is allowing major scientific advances. It is anticipated that the PMP will advance the field by facilitating similar research on diverse parasites and uncover patterns of microbial diversity and ecology that apply across phyla.

Parasite	Microbe(s)	Significance for health, agriculture, and/or the environment	References
*Opisthorchis viverrini*	*Helicobacter pylori* and other host gut bacteria	*O*. *viverrini* often leads to cholangiocarcinoma. Co-infection with oncogenic bacteria that are vectored towards the liver by the fluke may contribute to cancer development	[54–56]
*Trichomonas vaginalis*	TVV 1 through 3	Different clinical isolates of *T*. *vaginalis* show variable pathogenicity to the human host cells dependent on the TVV they carry; TVV released by dying and stressed parasites can explain why antibiotic therapy fails to prevent the inflammatory sequelae of parasitic infection	[57]
*Trichomonas vaginalis*	Host vaginal microbiome	Infection is detrimental to *Lactobacillus* and favors pathogenic bacteria associated with bacterial vaginosis	[58]
*Leishmania* spp.	LRV1	LRV1-infected *Leishmania* spp. increase severity of human leishmaniasis and lead to drug treatment failures	[59, 60]
Filarial nematodes	*Wolbachia*	Antibiotics, such as doxycyline and rifampicin, targeting the *Wolbachia* endosymbiont lead to loss of worm viability and fertility in human trials and increase antifilarial treatment efficacy	[61, 62]
Parasitoid wasps	Polydnaviruses and RNA viruses	Viruses contribute to parasitoid wasps virulence by modulating host immune response, host behavior, and feeding ability	[63–65]
Ticks	*Coxiella-*like endosymbiont	Symbiont codiversifies with its parasitic host and provides B vitamins missing from blood meals, enabling ticks to specialize in hematophagy	[66, 67]
*Vibrio shiloi*	Symbiotic zoonxanthellae of corals	*V*. *shiloi* produces toxins that target symbiotic zooxanthellae of the coral host inhibiting photosynthesis	[68]
*Trichuris* spp.	Host gut microbiome	The whipworm ingests bacteria from its direct environment and favors growth of mucolytic bacteria. Bacterial attachment is required for egg hatching	[69–72]
Digenetic trematodes including species of *Nanophyetes*, *Echinostoma*, *Fasciola*	*Neorickettsia* species	Endosymbiotic bacteria within cells of the trematode. These symbionts can be transferred horizontal from the trematode to mammalian host, where they are facultative pathogens	[73, 74]
*Pseudocapillaria tormentosa*	Zebrafish gut microbiota	Abundance of some bacteria taxa predicts helminth burden and intestinal lesions in host. Gut microbiome serves as diagnostic for parasite infection.	[75]

**Abbreviations:** LRV1, *Leishmania* RNA virus 1; PMP, Parasite Microbiome Project; TVV, *Trichomonas vaginalis* virus
